# Pd‐Lined Strained Trimetallic Au‐Ag‐Pd Nanoprism for Enhanced Electrocatalytic Activity Towards Formic Acid Oxidation

**DOI:** 10.1002/smsc.202500063

**Published:** 2025-04-15

**Authors:** Sourav Mondal, Sandip Kumar De, Tanmay Ghosh, Subrata Mondal, Mihir Manna, Dulal Senapati

**Affiliations:** ^1^ Chemical Sciences Division Homi Bhabha National Institute Saha Institute of Nuclear Physics A CI of Homi Bhabha National Institute 1/AF Bidhannagar Kolkata 700064 India; ^2^ Department of Chemistry SRICT‐ISR UPL University of Sustainable Technology Vataria Gujarat 393135 India; ^3^ Institute of Materials Research and Engineering (IMRE) Agency for Science, Technology and Research (A*STAR) 2 Fusionopolis Way, Innovis # 08‐03 Singapore 138634 Republic of Singapore; ^4^ Department of Chemistry Dinhata College Dinhata Cooch Behar 736135 India

**Keywords:** d‐band center, dehydrogenation pathway, lattice strain, synergistic effect, trimetallic heterostructure

## Abstract

Formic acid oxidation (FAO) reaction is an important electrocatalytic reaction in low‐temperature proton exchange membrane fuel cells. Pd‐based material has a superior electrochemical activity towards FAO. The activity of Pd‐based bimetallic materials is also well‐studied in the literature. Here, we have reported the synthesis of a unique heterostructured trimetallic nanoparticle where Pd is lined along with Ag forming a certain percentage of alloy at the edges of the bimetallic Au‐Ag prismatic nanotemplate. Though Pd acts as an effective material, this unique structure shows much improved catalytic activity due to the synergistic effect of Au, Ag, and Pd. Pd deposition increases the surface roughness and electrochemically active surface area. Lattice strain due to lattice mismatch between Ag and Pd modifies the d‐band center, enhancing the intrinsic activity, and facilitating the reaction kinetics. Pd‐deposited nanoparticle shows 3.4 and 4 times higher ECSA than monometallic cubic Pd nanoparticles and commercially available 10 wt% Pd/C. Our synthesized best catalyst Pd‐1.5 shows the mass activity of 634 Ag^−1^ which is ≈7 times higher than the standard 10 wt% Pd/C. Our catalyst shows higher stability and CO‐tolerance due to the suppression of the dehydration pathway and the reaction proceeds mainly via the dehydrogenation pathway.

## Graphical abstract

Thickness‐controlled Pd‐lined trimetallic Au‐Ag‐Pd nanoprism (Pd‐1.5) with modulated d‐band centre and increased lattice strain enhances the catalytic activity, ≈7 times compared to the standard 10 wt% Pd/C, towards the formic acid oxidation reaction (FAOR). Additionally, Pd‐1.5 shows higher stability and CO‐tolerance as the FAOR proceeds mainly via the dehydrogenation pathway by suppressing the dehydration pathway.

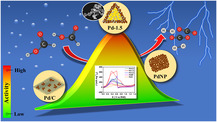

## 1 Introduction

Low‐temperature polymer electrolyte membrane (PEM) fuel cells can be operated with different anodic fuels such as hydrogen, methanol, ethanol, and formic acid (FA). There are some potential drawbacks for hydrogen and methanol as fuel like hydrogen is difficult to store and is highly flammable whereas methanol has innate toxicity, sluggish anodic oxidation kinetics, and fuel crossover problems in polymer electrolyte membrane. To avoid these problems formic acid can be a promising alternative fuel in PEM fuel cells with its characteristic properties such as inflammability at room temperature, fast electrooxidation, low fuel crossover through nafion, and ease of availability.^[^ ^1^, ^2^, ^3^ ^]^ Direct formic acid fuel cells (DFAFC) can be considered as one of the promising renewable energy sources with high energy conversion efficiency and low environmental pollution. Although the theoretical energy density of FA (2086 Wh L) is less than half of methanol (4690 Wh L), due to the lower rate of fuel crossover through the nafion membrane, a higher concentration of FA can be used which is desirable for designing compact portable power sources.^[^ ^4^, ^5^, ^6^, ^7^ ^]^ Moreover, the theoretical open circuit voltage (OCV) of direct formic acid fuel cells (DFAFCs) is 1.48 V, which is higher than other existing PEMFCs.^[^ ^8^ ^]^ Due to these advantages, formic acid is a potential source of energy as an anodic fuel in DFAFC if the generated CO_2_ is captured and recycled to achieve zero carbon emission. The anodic catalyst for the formic acid oxidation reaction (FAOR) largely determines the performance of DFAFCs. The decomposition of formic acid occurs via a dual pathway mechanism. In the dehydrogenation pathway hydrogen is released along with CO_2_ directly from the catalytic decomposition of HCOOH.^[^ ^9^ ^]^ On the other hand, CO is produced as an intermediate in the dehydration pathway, which is highly unwelcome and suppresses the FAOR activity.^[^ ^10^ ^]^

(1) $$\text{Dehydrogenation pathway}:\text{HCOOH}\to {\text{CO}}_{2}+2H^{+}+2e^{-}$$

(2) $$\text{Dehydration pathway}:\text{HCOOH}\to {\text{CO}}_{\text{ads}}+H_{2}O\to {\text{CO}}_{2}+{\text{2H}}^{+}+2e^{-}$$

So, developing highly active and selective electrocatalysts for dehydrogenation pathways is an important and active topic of renewable energy research. Platinum has been widely used as an electrocatalyst because it has the highest electrocatalytic activity among the anode metal catalysts for the electrooxidation of small organic fuels. However, the performance of the catalyst decreases as the surface of platinum is heavily poisoned by the strong adsorption of CO intermediate produced during the oxidation of organic fuels due to the strong Pt—CO bond.^[^ ^11^ ^]^ Because of the limited abundance and enhanced CO‐poisoning of platinum, recent extensive research efforts have been devoted to the development of non‐platinum electrocatalysts with comparable catalytic activities. Pd was identified as one of the most effective electrocatalysts for formic acid electrooxidation as the reaction kinetics is faster on Pd and it shows excellent anti‐poisoning properties resulting from the high selectivity of Pd towards dehydrogenation process over the dehydration process during the oxidation of formic acid.^[^ ^4^, ^12^, ^13^, ^14^ ^]^ Another advantage of Pd over Pt is that FAOR on Pt typically proceeds via two oxidation pathways parallelly whereas on Pd it generally follows through a dehydrogenation pathway selectively.^[^ ^15^ ^]^ However, the activity and stability of monometallic Pd still require improvement for it to be used as an effective catalyst in formic acid fuel cells. Moreover, the recent increase in price, limits the use of Pd as a catalyst in FA oxidation. To handle these difficulties different strategies have been used such as alloying Pd with other metals, core‐shell structure formation, use of carbon support, etc. through modifying electronic properties and geometrical structures. For example, Tang and co‐workers have shown the enhanced activity and stability of Pd‐Ag alloy towards FAOR.^[^ ^16^ ^]^ Yang and co‐workers have shown that Au_core_@Pd_shell_ shows high FAOR performance due to increased lattice strain and electrochemical surface area.^[^ ^17^ ^]^ The electrocatalytic activity of carbon‐supported bi‐ and tri‐metallic catalysts towards FAOR has been shown by Su Ha and his co‐workers.^[^ ^12^ ^]^ Interestingly they have shown that trimetallic catalysts have higher activity than the corresponding mono and bimetallic catalysts. In recent years, research into catalytic applications has highlighted the advantages of using multi‐metallic nanoparticles over their monometallic and bimetallic counterparts.^[^ ^18^, ^19^ ^]^ The enhanced catalytic activity of multi‐metallic nanoparticles can be attributed to several key factors like i) Synergistic effect: where the properties of individual metals complement and enhance each other. This can improve the overall catalytic performance.^[^ ^20^, ^21^ ^]^ ii) Optimized Electronic Properties: where the presence of multiple metals can modify the electronic structure of the nanoparticles, leading to improved charge transfer and adsorption characteristics. This can result in better binding of reactants, thereby accelerating chemical reactions.^[^ ^22^ ^]^ iii) Increased Active Sites: multi‐metallic nanoparticles can increase the number of active sites available for catalytic reactions. Each metal may offer unique active sites that can activate different pathways, enhancing the overall catalytic efficiency.^[^ ^23^ ^]^ iv) Improved Stability: Multi‐metallic nanoparticles can exhibit enhanced thermal^[^ ^24^ ^]^ and structural^[^ ^25^ ^]^ stability compared to pure metals or bimetallic systems. This can lead to prolonged catalytic activity and reduces the need for frequent catalyst replacement. Several research groups have shown enhanced catalytic activity of trimetallic nanoparticles for formic acid oxidation reaction.^[^ ^26^, ^27^, ^28^, ^29^ ^]^

In this article, we have successfully synthesized a heterostructured trimetallic (Au‐Ag‐Pd) nanoparticle where the Au‐Ag forms a triangular template. Because of the lower standard reduction potential of Ag^+^/Ag (+0.7991 V versus SHE) than that of Pd^2+^/Pd (+0.915 V), galvanic replacement reaction provides an effective route to synthesize heterostructured Pd‐Ag bimetallic nanomaterials by using Ag nanoparticle as template with different morphology and surface arrangement.^[^ ^30^ ^]^ In this work, on the Au‐Ag template, we have deposited Pd of different thicknesses which gives the unique shape and arrangement of Pd/Ag atoms to form the heterostructure. Though Pd acts as the effective material for HCOOH oxidation, here both Au and Ag play a crucial role in tuning the adsorption and desorption energy and enhancing the catalytic activity. The as‐prepared Pd‐deposited Au‐Ag heterostructure exhibits excellent electrocatalytic activity towards FAO.

## 2 Experimental Section

### 2.1 Chemicals

Chemicals including gold(III) chloride trihydrate (HAuCl_4_·3H_2_O; ≥99.9%, trace metal basis), silver nitrate (AgNO_3_; BioXtra, ≥99%, titration), palladium(II) chloride (Reagent Plus, 99%), 5% Nafion solution (Merck), sodium borohydride (NaBH_4_; granular, 10–40 mesh, 98%), L‐ascorbic acid (C_6_H_8_O_6_; ACS Reagent, 99%), sodium citrate tribasic dihydrate (Na_3_C_6_H_5_O_7_·2H_2_O; ≥99.5%, NT), hexadecyltrimethylammonium bromide or CTAB (C_19_H_42_BrN; BioUltra, for molecular biology, ≥99.0% (AT)), and formic acid (ACS, 98–100%) were purchased from Sigma‐Aldrich, India, and used without any further purification.

### 2.2 Material Synthesis

A three‐step synthetic procedure was used to synthesize the trimetallic heterostructure. In the 1st step, the silver seed was synthesized. In a typical synthetic procedure, 200 μL of 2.5 × 10^−2^ m trisodium citrate (TSC) is added to 20 mL milli‐Q water in a 50 mL conical flask. Then 500 μL of 10^−2^ m AgNO_3_ was added followed by the addition of freshly prepared ice‐cold 60 μL of 10^−1^ m NaBH_4_ under a constant stirring (200 rpm). The colour of the solution immediately turns into a bright yellow colour which confirms the formation of silver nanoseed. The solution is kept in the dark for two hours.

In the 2nd step, bimetallic nanoprism is synthesized following the procedure described by De et al.^[^ ^31^ ^]^ In a typical growth procedure, 0.5 g of CTAB was dissolved in 45 mL water at 30 °C under sonication. Then 2 mL of 10^−2^ m of AgNO_3_ was added to the solution under steady stirring conditions (200 rpm). Next, 300 μL of HAuCl_4_ (10^−2^ m) is added followed by the addition of 320 μL of 10^−1^ m freshly prepared ascorbic acid. The addition of gold solution and ascorbic acid is repeated once again. The addition of ascorbic acid causes the yellow color of the gold solution to turn colorless indicating the reduction of Au(III) ions. Immediately 250 μL of the pre‐synthesized silver seed solution is added and homogenized for 15 s and kept the resultant solution undisturbed overnight.

**Scheme 1:**
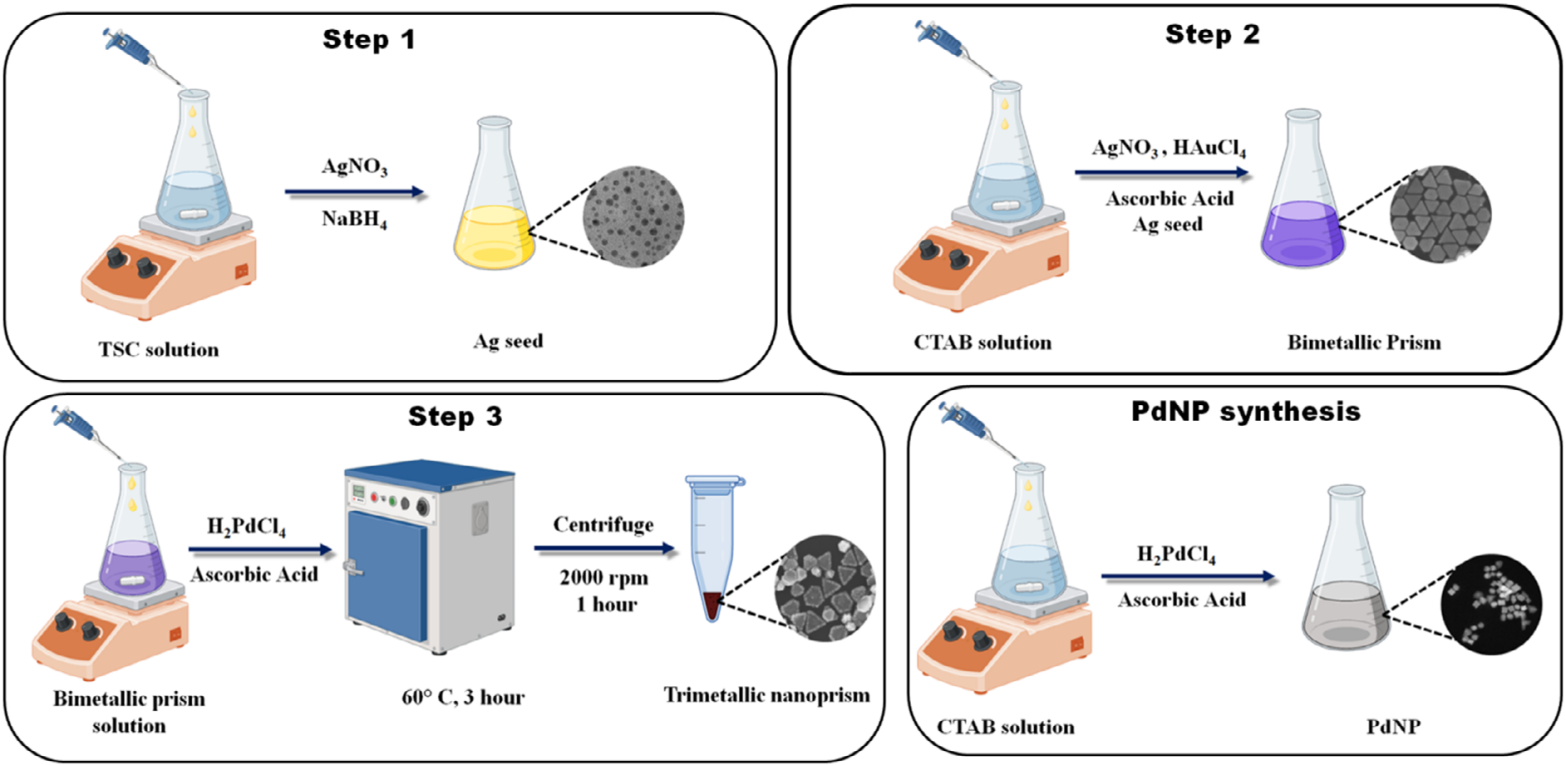
A schematic presentation of a three‐step synthesis of trimetallic heterostructure along with one‐step synthesis of monometallic PdNP.

In the 3rd step, we have deposited palladium on the bimetallic prism as synthesized in the 2nd step. In the synthetic procedure, 25 mL of 3 mM CTAB solution was taken in a 50 mL conical flask. Then the entire volume of the centrifuged bimetallic prism is dispersed into this CTAB solution under constant stirring. Next, 500 μL of 10^−1^ m ascorbic acid is added followed by the addition of palladium solution (For the preparation of 10 mM H_2_PdCl_4_, we dissolved 44.6 mg PdCl_2_ in 25 mL 0.02 m HCl by sonication at 70 °C) of different amounts (500, 1500, and 2500 μL) for varying thickness of palladium layer. Finally, the resultant solution (in the conical) is transferred into an oven and kept at 60 °C for 3 h. The color of the solution becomes dark brown to black depending on the amount of Pd^2+^ added to the solution as elaborated in **Scheme** **1**.

To compare the catalytic activity of a Pd‐based trimetallic nanoparticle with the monometallic Pd counterpart, a cubic palladium nanoparticle (PdNP) was prepared following a similar procedure for Pd deposition without adding the bimetallic prism template. In brief, 25 mL of 3 mM of CTAB was taken in a 50 mL conical flask, and to that we added 1500 μL of 10 mM H_2_PdCl_4_ solution and after a proper homogenization, we reduced Pd(II) by using 500 μL of 10^−1^ m ascorbic acid. Finally, the resultant mixture is similarly transferred into the same oven and kept at 60 °C for 3 h. The color of the solution changes from light yellow to dark brown indicating the formation of Pd cubes.

A bimetallic Au‐Ag triangular particle without Pd deposition is named Prism. While, 500, 1500, and 2500 μL Pd deposited particles are named as Pd‐0.5, Pd‐1.5, and Pd‐2.5 respectively. The monometallic cubic palladium particle is named PdNP throughout this report.

### 2.3 Material Purification

After the complete growth of the prisms overnight the samples were centrifuged at 2000 rpm for 1.5 h. The collected particles were redispersed in 30 mL milli‐Q water and centrifuged again at 2500 rpm for 1 h. After palladium deposition, Pd‐0.5, Pd‐1.5, and Pd‐2.5 particles were all centrifuged at 2000 rpm for 1 h and finally collected each centrifuged particle with a total volume of 500 μL. PdNP is collected by centrifuging the solution at 5000 rpm for 1 h and collects a total volume of 500 μL. All the particles were washed two times to remove excess surfactant and unnecessary ions.

### 2.4 Material Characterization

UV‐Vis spectra were recorded on a Jasco‐V770 spectrophotometer using a 1 cm path‐length quartz cuvette to confirm the plasmonic nature of the nanoparticles. Scanning electron microscopy (SEM) images were obtained using a Zeiss Supra 40 field‐emission scanning electron microscope. The morphology and size of the particles were characterized by transmission electron microscopy (TEM) and high‐resolution TEM (HRTEM) measurements on an FEI Tecnai G2F30 S‐Twin microscope (300 kV). The compositional analysis and elemental mapping were performed by energy‐dispersive X‐ray spectroscopy (EDX) attached to the Tecnai G2 F30 S‐Twin microscope. X‐ray diffraction (XRD) patterns were obtained using a Rigaku Smart Lab X‐ray diffractometer with Cu Kα1 radiation. The 2*θ* range for the measurement was set from 20°–90°. The surface properties and electronic structure of the nanoparticles were performed by X‐ray photoelectron spectrometer (XPS) measurements on an Omicron Multiprobe (Omicron NanoTechnology GmbH, UK) spectrometer fitted with an EA‐125 hemispherical analyzer.^[^ ^32^ ^]^ Each sample was drop casted on a *p*‐type silicon wafer and dried overnight in a vacuum before XPS measurement. The concentration of the individual elements in solution for each nanostructure was measured by an inductively coupled plasma optical emission spectrometer (ICP‐OES, ICAP DUO 6500 from Thermo Fisher).

### 2.5 Electrochemical Characterization

Electrochemical measurements of these as‐prepared catalysts were performed on a CHI 6092E electrochemical workstation (CH Instruments, USA) in a three‐electrode one‐compartment cell setup. A conventional three‐electrode system was used with a modified glassy carbon electrode (3 mm diameter) as the working electrode, an Ag/AgCl (Saturated KCl) electrode as the reference electrode, and a Pt rod as the counter electrode. Before each experiment, the glassy carbon electrode was first polished with alumina powders (1, 0.3, 0.05 μm) and washed followed by sonication in an ethanol‐water mixture to obtain a mirror finish. To prepare the catalyst‐coated GC electrode,10 μL of catalyst (catalyst loading for Pd‐0.5: 5.14 μg, Pd‐1.5: 17.70 μg, Pd‐2.5: 41.79 μg, and PdNP: 16.92 μg) is mixed with 5 μL of 1.6% Nafion (polymeric binder) solution and the resulting solution is drop casted and air dried. To prepare the Pd/C catalyst slurry, a weighted amount of the catalyst is mixed with 660 μL milli‐Q water, 320 μL isopropanol, 20 μL Nafion, and sonicated for 1 h. A 10 μL of the slurry was dropcasted for comparison. 0.5 m H_2_SO_4_ was used as an electrolyte and deaerated the electrolyte by bubbling N_2_ through the electrolyte for 20 min. All experiments were performed at a temperature of 25 ± 1 °C. The electrochemical performance of formic acid oxidation was evaluated by cyclic voltammetry (CV) and chronoamperometry (CA). CV measurements were performed in a mixture of 0.5 m HCOOH and 0.5 m H_2_SO_4_ at a scan rate of 50 mV s^−1^. CA measurements were performed at a constant potential of 0.1 V (vs. RHE) for 3600 s. CO stripping voltammetry was performed in the following procedure. First, 0.5 m H_2_SO_4_ electrolyte was saturated with CO by purging CO gas for 20 min. The electrode was immersed into the solution under a constant potential of 0.05 V (potential close to the thermodynamic potential of oxidation of CO) for 600 s to form a CO adsorption layer on the surface of the catalyst. Then N_2_ was purged into the solution for 20 min to remove the dissolved CO in the solution. Later adsorbed CO was stripped by CV in the potential region of 0–1.4 V at a scan rate of 50 mV s^−1^ in CO‐free electrolyte.

## 3 Result and Discussion

### 3.1 Optical and Structural Analysis of the Nanoparticle

UV‐Vis absorption spectra of the bimetallic prism and trimetallic particle are shown in (Figure S1A, Supporting Information). The bimetallic prism shows two prominent plasmonic bands around 550 nm and 780 nm and a small hump at ≈370 nm. The appearance of the hump near 370 nm is due to the *out‐of‐plane* quadrupole resonance, the surface plasmon band near 550 nm originates from *out‐of‐plane* dipole resonance (transverse mode), and *in‐plane* dipole resonance (longitudinal mode) arises at 780 nm for our synthesized bimetallic prism.^[^ ^33^, ^34^, ^35^ ^]^ Pd nanoparticles as such have no plasmonic band in the UV‐Vis region.^[^ ^36^, ^37^ ^]^ So from the figure, it is evident that as more and more Pd is getting deposited on the bimetallic prism template, it suppresses the surface plasmon of gold and silver and the plasmon intensity gradually decreases from Pd‐0.5–Pd‐2.5. This observation indicates an effective coating of palladium on bimetallic prism particles.

The compositional analysis of the synthesized nanoparticles is shown by ICP‐OES data in **Table** **1**. From the values, it can be seen that the amount of Au and Ag remains almost constant for all the synthesized particles while the amount of Pd increases gradually with the increase of Pd precursor salt added during the synthesis of different trimetallic nanoparticles.

**Table 1:** ICP‐OES data for different nanostructures used for this study.

ICP‐OES data			
Nanoparticle	Au [mg mL]	Ag [mg mL]	Pd [mg mL]
Prism	1.758	0.331	–
Pd‐0.5	1.908	0.318	0.514
Pd‐1.5	1.701	0.375	1.770
Pd‐2.5	1.878	0.381	4.179
PdNP	–	–	1.692

**Figure 1:**
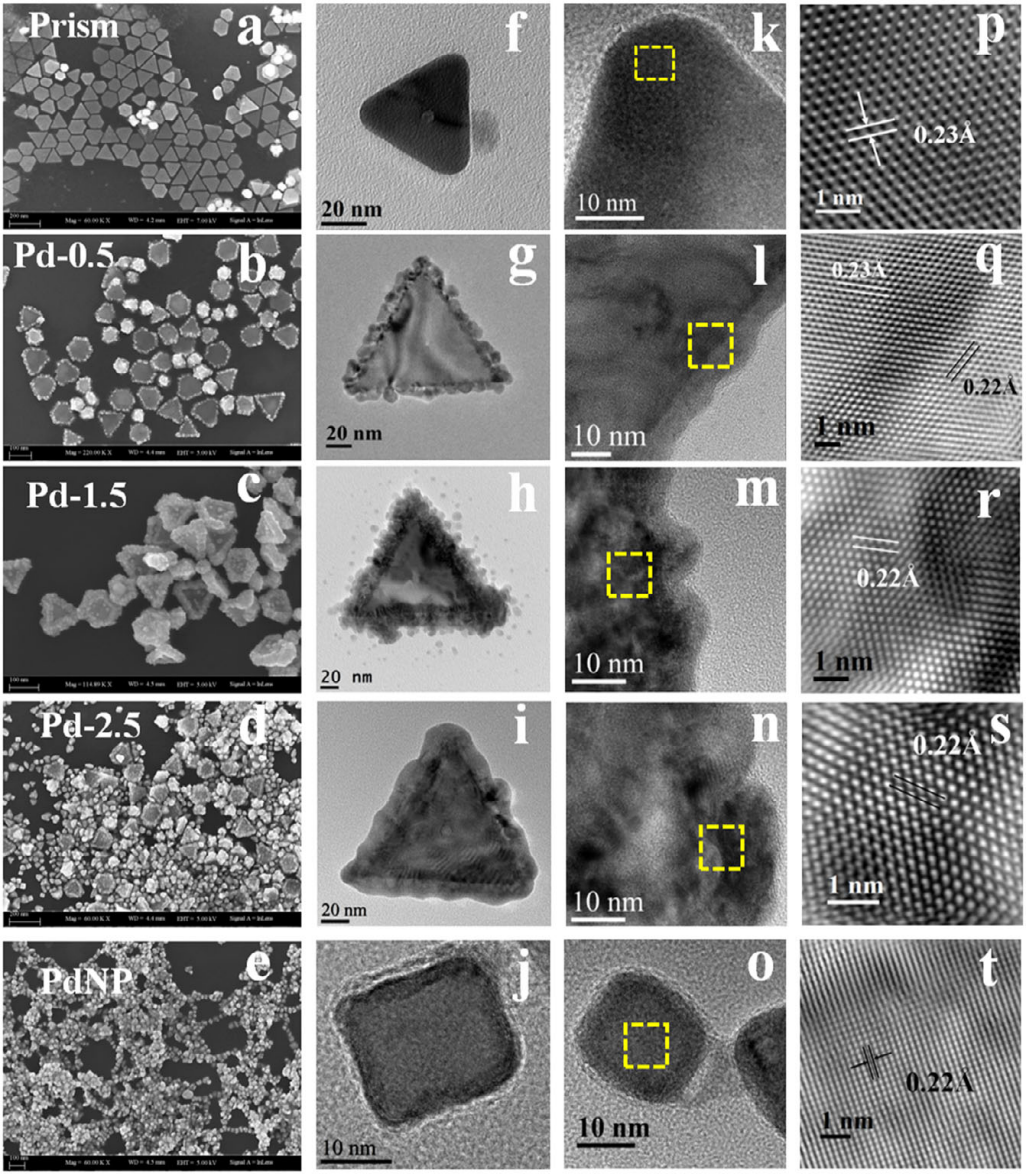
a–e) SEM images, f–j) TEM images, k–o) HRTEM images, and p–t) IFFT images of the bimetallic prism, Pd‐0.5, Pd‐1.5, Pd‐2.5, and PdNP, respectively.

**Figure** **1**a–e shows the SEM image of the bimetallic prism, Pd‐0.5, Pd‐1.5, Pd‐2.5, and pure PdNP respectively. In the case of Pd‐0.5, it is evident from the above image that a thin layer (≈14 nm) of Pd is deposited at the edges of the bimetallic prism. In the case of Pd‐1.5, the deposition of the palladium layer increases to ≈22 nm. With further increase of Pd precursor salt, the thickness of Pd deposition (≈25 nm) doesn't increase much for Pd‐2.5 but some other irregular shapes of particles have been formed as is clear from Figure 1d. At such high concentrations, Pd itself forms nucleation centres and starts to grow Pd nanoparticles along with galvanic replacement of Ag (described later in detail) in the bimetallic template. When the same protocol for Pd deposition is used without adding a bimetallic prism, it forms a cubic‐shaped Pd nanoparticle of size ≈24 nm. Figure 1f–j show the TEM images of the bimetallic, trimetallic, and monometallic nanoparticles respectively. The average edge lengths of Pd‐0.5, Pd‐1.5, and Pd‐2.5 are 120 ± 2.5, 142 ± 3.9, and 151 ± 4.2 nm respectively. Figure 1k–t show the corresponding HRTEM image and IFFT of the selected region. In the bimetallic prism, the d‐spacing is 0.23 Å corresponding to the (111) plane of FCC Au or Ag. We have also identified the presence of high energy diffraction spots which can be indexed to 1/3(422), (422), and (220) facets which are clearly observable from our recorded SAED pattern, included in the as (Figure S1B, Supporting Information). The d‐spacing of pure PdNP is 0.22 Å which represents the (111) plane of the FCC Palladium crystal lattice. In Pd‐0.5 particles, two types of lattice planes are identified at two different regions that correspond to the Pd as the outer layer (d‐spacing: 0.22 Å) on the Au/Ag bimetallic prism (d‐spacing: 0.23 Å). From the d‐spacing, a dominating Pd deposition at the edges of the bimetallic prism is confirmed. Sharp edges and truncated corners in the resultant trimetallic nanoparticle contain plenty of low‐coordinated sites compared to smooth spherical nanoparticles. Additionally, the abundance of stepped regions at the interface between the Pd‐layer and Au‐Ag basal plane which is shown in (Figure S1C, Supporting Information) also act as catalytically active sites.

**Figure 2:**
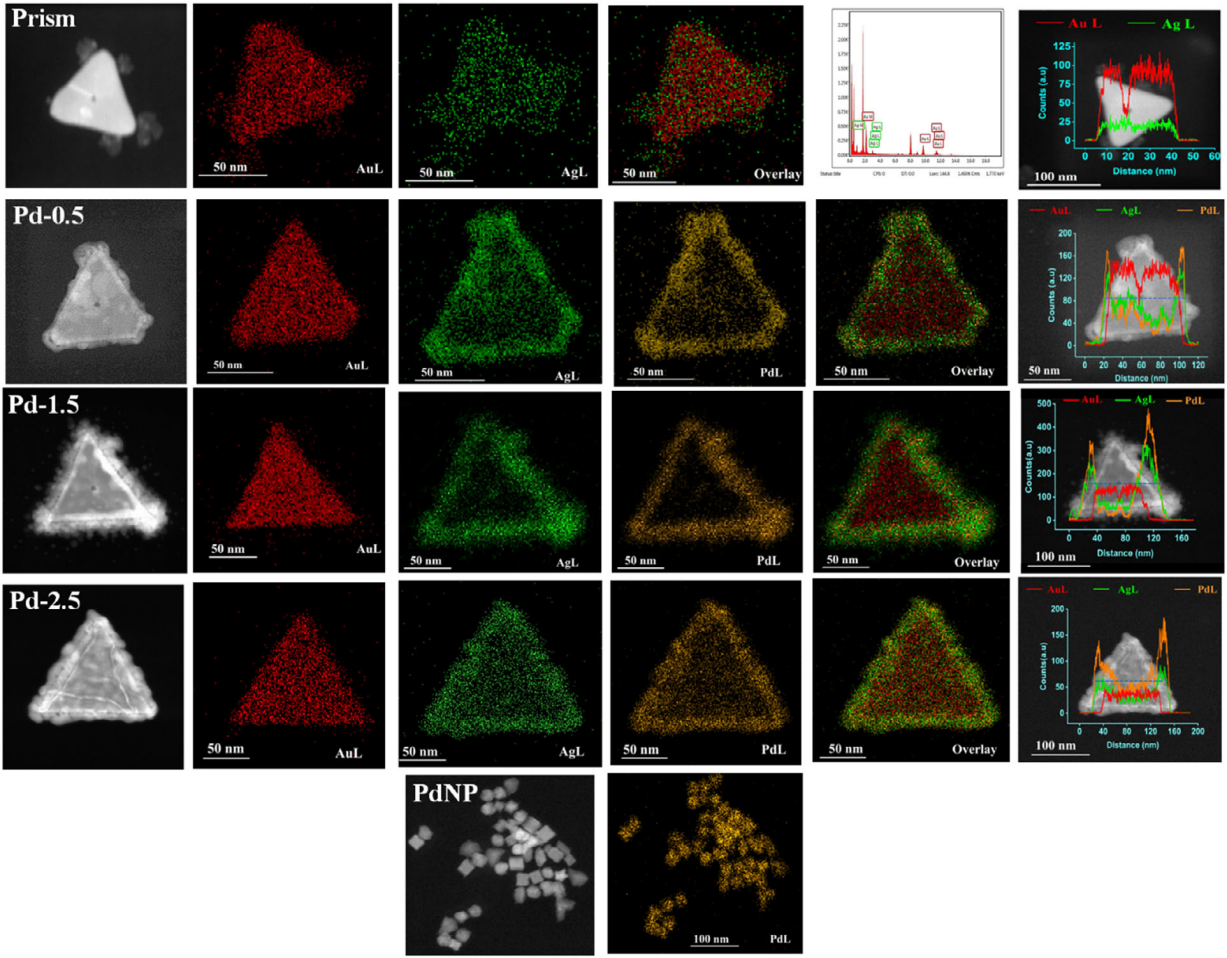
HAADF, EDX elemental mapping, and EDX line profile of the bimetallic prism (1st row), Pd‐0.5 (2nd row), Pd‐1.5 (3rd row), Pd‐2.5 (4th row), and PdNP (5th row).

**Scheme 2:**
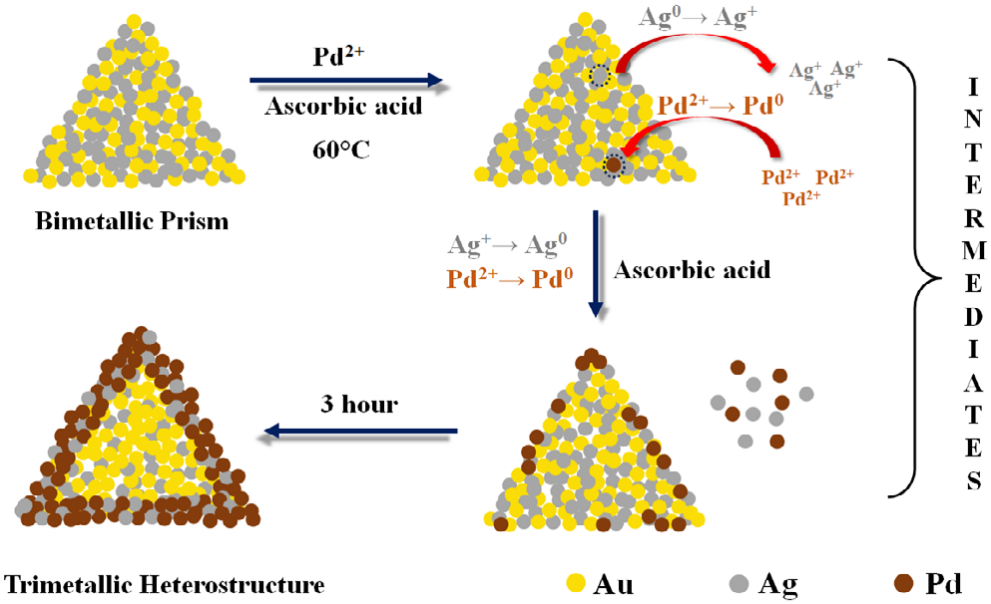
Schematic representation shows the mechanistic details of the co‐deposition of Pd and Ag on the edges of the Au‐Ag prism.

The spatial and orientational distribution of Au, Ag, and Pd in the Pd‐Ag‐Au heterostructure was revealed by high‐angle annular dark‐field scanning TEM (HAADF‐STEM), EDX elemental mapping pattern, and EDX line scanning profile. For the bimetallic prism structure, it is clearly observed that Au and Ag both are present throughout the prism structure and form an alloy as there is no clear boundary between the two elements. The EDX line profile of bimetallic prisms shows the presence of a higher amount of Au (68%) compared to Ag (32%). A predominant dip in Au counts and a slight enhancement in Ag count at the center of the prism represents the position of the Ag‐seed in bimetallic nanoprism. In the case of Pd‐deposited particles, an interesting point is noticed. From the color mapping of Pd‐0.5, Pd‐1.5, and Pd‐2.5 particles it is observed that not only Pd but Ag also gets deposited at the edges of the prism along with Pd. Line profile also gives a similar observation. The EDX line profile of Pd‐1.5 shows the atomic percentages of Au (≈8%), Ag (≈35%), and Pd (≈57%). **Figure** **2** not only shows us the spatial distribution of different metals on nanoprism but also informs us about the mechanism of particle formation. It is quite interesting to note that though Ag and Pd are deposited exactly at the same position, they do not form a 100% alloy structure which is confirmed by XRD (described later in detail). So there may be a co‐existence of Ag and Pd particles in the same region. As the reduction potential of Pd (Pd^2+^ + 2e^−^ → Pd, *E* ^0^ = 0.91 V versus SHE) is higher than Ag (Ag^+^ + e^−^ → Ag, *E* ^0^ = 0.79 V versus SHE), when we add palladium precursor salt to the solution of bimetallic prism template, the Ag atoms are leached out by Pd^2+^ as Ag^+^, and Pd^2+^ is self‐reduced to Pd^0^.^[^ ^38^ ^]^ As the reaction medium contains ascorbic acid as a reducing agent at 60 °C, the oxidized Ag^+^ and residual Pd^2+^ are simultaneously co‐reduced to form the resultant structure which contains both Ag and Pd atoms at the edges of the prism with Au forming the triangular base structure. To confirm the gradual co‐deposition of Ag^0^ and Pd^0^, we have measured the thickness of the edge, containing both Ag^0^ and Pd^0^, as a function of time as shown in (Figure S2, Supporting Information). It is clearly demonstrated in (Figure S2, Supporting Information) that the thickness of the Pd‐Ag layer increases from 10.6 nm after 4 min–13.7 nm after 10 min and 19.1 nm after 30 min of the Pd addition in the 3rd step of the reaction. Since the bimetallic prism contains (110) facets on the edges and the relative surface energy of the different crystal facets are in the order *γ*(111) < *γ*(100) < *γ*(110), initially Pd starts to deposit on the edges of the bimetallic prism^[^ ^39^ ^]^ to stabilize the (110) facet. The mechanistic representation of the co‐deposition of Pd and Ag on the edges of a bimetallic prism is shown in **Scheme** **2**. To check the effect of temperature we have also carried out the synthesis of Pd‐1.5 heterostructure at room temperature (27 °C) and the corresponding EDS colour mapping of the synthesized nanoparticle is shown along with its HAADF image in (Figure S3, Supporting Information). The obtained atomic percentage of three different elements (Au, Ag, and Pd) in Pd‐1.5 synthesized at room temperature is ≈3%, ≈8%, and ≈89%. Since the reduction capability of AA increases with temperature (more availability of nascent hydrogen), at elevated temperature (60 °C) AA substantially reduces the leached‐out Ag^+^ to Ag^0^ and thereby enhances the metallic silver deposition on Pd‐1.5 nanoparticle which is clearly observable from the measured atomic percentage of Au, Ag, and Pd as ≈8%, ≈35%, and ≈57% respectively (Figure 2).

**Figure 3:**
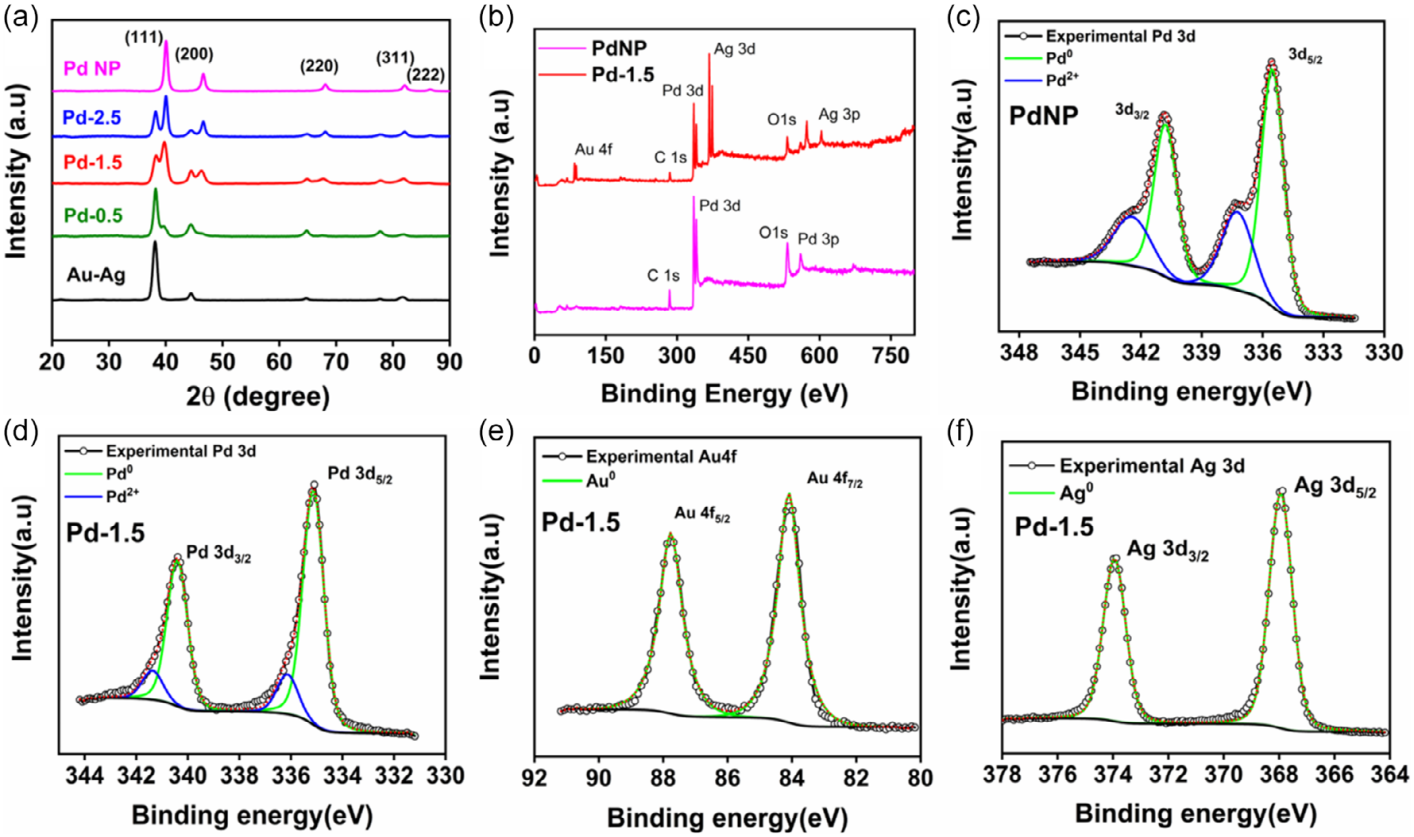
a) XRD pattern of the bimetallic prism, Pd‐0.5, Pd‐1.5, Pd‐2.5 and PdNP; b) XPS Survey spectra of PdNP and Pd‐1.5; c) XPS spectrum of Pd3d for PdNP; and XPS spectrum of d) Pd3d, e) Au4f, f) Ag3d for Pd‐1.5.

In **Figure** **3**a the X‐ray diffraction pattern of bimetallic prism shows the formation of (111), (200), (220), (311), and (222) planes corresponding to the 2*θ* values of 38.1°, 44.4°, 64.6°, 77.6°, 81.7° respectively which confirms the formation of an FCC lattice structure of Au and Ag [JCPDS No.04‐0783 (Ag) and JCPDS No.04‐0784 (Au)]. In the case of pure PdNP, five peaks located at 40.04°, 46.6°, 68.08°, 82.08°, and 86.56° can be indexed to (111), (200), (220), (311), (222) FCC Pd lattice respectively (JCPDS No.46‐1043 (Pd)). For the Pd deposited NPs, XRD spectra show peaks corresponding to Pd along with gold and silver lattice. Since Pd and leached‐out Ag atoms are co‐reduced in the particle formation process, there is a possibility of alloy formation. Since Au isn't involved in the co‐reduction process, the XRD peak position corresponding to the Au FCC lattice remains unaltered. In the case of Pd‐1.5 two sets of peaks are observed for Au/Ag and Pd. The diffraction peaks at the 2*θ* value 39.7°, 46.2°, 67.7°, and 81.6° could be indexed to Pd (111), (200), (220), (311) planes.^[^ ^40^ ^]^ A slight shift in the diffraction peak of Pd towards a lower 2*θ* value due to the incorporation of silver into the palladium lattice suggests the formation of silver–palladium alloy. To calculate the percentage of alloying in Pd‐1.5 particle the Pd(111) peak is used to analyze the lattice constant, a, by using Vegard's law.

(3) $$a=\frac{\surd 3\lambda_{K_{\alpha 1}}}{2\text{sin}\theta }$$

Where $\lambda_{K_{\alpha 1}}$ = 1.54056 nm. According to the values of the lattice constant, we can calculate the percentage of alloying of Ag in Pd using the equation $a=a_{0}+kx_{\text{Ag}}$, where $a_{0}=3.896$ Å is the lattice constant of pure PdNP and $x_{\text{Ag}}$ is the fraction of Ag in the alloy form. The lattice constant ‘a’ was calculated for Pd‐1.5 particle using Pd(111) peak is 3.962 Å. The constant *k* = 0.189 is the difference of lattice constants between Ag and Pd as calculated from Vegard's law. By knowing ‘*a*’, ‘*a*_0_’, and *k*, we can easily calculate the value of $x_{\text{Ag}}$. The obtained percentage of Ag atoms in the alloy state is calculated as 16.7% which explains that only a small fraction of Ag is involved in alloying and the remaining Ag atoms remain in their original FCC structure.^[^ ^41^ ^]^ Finally, the generated strain in the Pd‐1.5 nanoparticle is calculated using the formula.

(4) $$\text{Strain}=\frac{a-a_{0}}{a_{0}}\times 100\%$$

Where ‘*a*’ and ‘*a*_0_’ are the deviated and standard lattice parameters respectively. The lattice strain for Pd‐1.5 is calculated as 1.8% compared to 0.07% of Pd‐2.5 which explains its enhanced catalytic activity.

XPS was performed to characterize the chemical and surface electronic properties of nanoparticles. The survey spectrum of PdNP and Pd‐1.5 is shown in Figure 3b which confirms the presence of Pd in PdNP and Au, Ag, and Pd in the synthesized Pd deposited particle (Pd‐1.5). (Figure S4a, Supporting Information) shows the high‐resolution C 1s XPS spectrum with three different components. The most intense peak at the binding energy (BE) of 284.6 eV corresponds to sp^3^ hybridized C—C species. The other two peaks at the BEs of 285.2 and 286.6 eV are assigned to C=C (sp^2^), and C—O species respectively. Figure 3c shows the XPS spectrum of monometallic PdNP where the Pd3d can be fitted by two pairs of overlapping Lorentzian‐Gaussian curves in terms of the metallic and oxidized state. The peaks at 335.5 and 340.7 eV are attributed to metallic Pd^0^ 3d_5/2_ and Pd^0^ 3d_3/2_ respectively while other pairs of peaks at 337.2 and 342.5 eV are ascribed as oxidized Pd i.e., Pd(II)3d_5/2_ and Pd(II)3d_3/2_ respectively. For Pd‐1.5 particles the XPS spectrum of Pd3d, Au4f, and Ag3d are shown in Figure 3d–f. The observed energy difference between the 3d_5/2_ and 3d_3/2_ peaks for Ag is 6.0 eV, while for Pd, the difference between the 3d_5/2_ and 3d_3/2_ peaks is 5.3 eV. For Au, the energy difference between the 4f_7/2_ and 4f_5/2_ peaks is 3.7 eV. These values were the same as the reported values for zero‐valent Ag, Pd, and Au respectively.^[^ ^42^ ^]^ In Figure 3d two major peaks at BE of 335.1 and 340.4 eV can be assigned to Pd^0^ 3d_5/2_ and Pd^0^ 3d_3/2_ spin‐orbit ground and excited states respectively. In addition, two minor peaks at 336.1 and 341.3 eV correspond to Pd^2+^ 3d_5/2_ and Pd^2+^ 3d_3/2_, respectively, were observed, indicating not only the presence of a smaller amount of surface Pd oxide (Pd^2+^) species but also a higher percentage of the spin‐orbit ground state in both the oxidation state. Notably, the binding energy for Pd3d core levels shifted slightly to a lower value compared to the monometallic PdNP. Such shifting of binding energy is indicative of charge transfer between contacting Pd and Ag atoms due to the formation of alloy structure.^[^ ^43^, ^44^, ^45^ ^]^ One interesting point to note here is that monometallic palladium nanoparticles are more prone to form oxide due to aerial oxidation than palladium particles in the trimetallic heterostructure which is visible from the area under the curve comparison for PdNP and Pd‐1.5 (for PdNP, Pd^0^:Pd^2+^ = 2.1:1 and for Pd‐1.5, Pd^0^:Pd^2+^ = 5.5:1). So alloying with Ag in the heterostructure certainly gives the desired stability to the Pd particle in its heterostructure which would benefit its catalytic activity. We have checked the XPS spectra of Pd‐1.5 after 8 h of chronoamperometric measurement in 0.5 m H_2_SO_4_ medium with 0.5 m HCOOH as shown in (Figure S4, Supporting Information) (d–f) for Au, Ag, and Pd respectively. We have seen two peaks each for Au and Ag corresponding to their spin‐orbit ground (Ag: 3d_5/2_ and Au: 4f_7/2_) and excited state (Ag: 3d_3/2_ and Au: 4f_5/2_) solely for metallic Au and Ag. Moreover, by comparing Figure 3e with S4d, Supporting Information, and Figure 3f with S4e, Supporting Information, it is clear that there is apparently no shift in binding energy both for Au and Ag after 8 h of chronoamperometric reaction which is also elaborated in **Table** **2**. As shown in (Figure S4f, Supporting Information) two major peaks at BE of 335.6 and 340.9 eV for Pd^0^ 3d_5/2_ and Pd^0^ 3d_3/2_ spin‐orbit ground and excited states shift nominally to the higher binding energy value than the Pd‐1.5 particle before reaction. Moreover, the Pd^0^:Pd^2+^ = 4.8:1 indicates the superior stability of the particle as most of the Pd retain in their metallic state even after 8 h of continuous reaction. Due to a small amount of charge transfer from the overall Pd particle, BE shifts towards a higher value and the amount of oxidized Pd increases slightly. In Figure 3e, two peaks appeared at 84.0 and 87.7 eV belonging to spin‐orbit ground (Au4f_7/2_) and excited (Au4f_5/2_) states, respectively. It is also noticed by comparing Figure 3e with (Figure S4c, Supporting Information) that there is no change in binding energy for Au after the Pd deposition on bimetallic nanoprism (Table 2). In Figure 3f peaks located at 367.9 and 373.9 eV are attributed to spin‐orbit ground (Ag3d_5/2_) and excited (Ag3d_3/2_) states of metallic silver (Ag^0^) of Pd‐1.5 particle. XPS spectra of the silver component (Ag3d) in the bimetallic prism are shown in (Figure S4b, Supporting Information). In this figure, four prominent peaks are identified for different spin‐orbit redox states. Peaks located at 368.4 and 374.4 eV belong to the spin‐orbit ground (Ag3d_5/2_) and excited (Ag3d_3/2_) state of metallic silver (Ag^0^) whereas peaks located at 367.5 and 373.5 eV belong to the oxidized silver (Ag^+^). Quantitative analysis of peaks indicates that the concentration of Ag^+^ is higher than that of Ag^0^ (Ag^+^:Ag^0^ = 1.2:1). This Ag^+^ is counterbalanced by Br^−^ ion form CTAB and stabilizes the low coordinated atomic sites. When Pd is introduced and it forms an alloy with Ag, the BE of Ag^0^ shifts to a lower BE value as shown in Figure 3f and listed in Table 2. This substantial shift in BE (≈0.5 eV) proves a dominating transfer of electron density from Ag to Pd as the electronegativity of Pd is higher than Ag (In Pauling scale χ_Pd_ = 2.20 and χ_Ag_ = 1.93). Another interesting point to note is that due to the incorporation of Pd, no significant peak is observed for oxidized silver. This again proves our proposed hypothesis of trimetallic nanoparticle formation i.e., oxidized Ag gets reduced in the 3^rd^ step of the reaction where ascorbic acid is added as a reducing agent at 60 °C and deposits as metallic silver along with Pd.

**Table 2:** Binding energy of different zero valent spin‐orbit states of metal elements present in different nanoparticles, obtained from XPS studies.

Nanoparticle	Au 4f_5/2_	Au 4f_7/2_	Ag 3d_3/2_	Ag 3d_5/2_	Pd 3d_3/2_	Pd 3d_5/2_
Prism	87.6	84.0	374.4	368.4	–	–
PdNP	–	–	–	–	340.7	335.5
Pd‐1.5	87.7	84.0	373.9	367.9	340.4	335.1
Pd‐1.5 (after 8 h reaction)	87.8	84.1	373.7	367.7	340.9	335.6

### 3.2 Electrochemical Test

**Figure 4:**
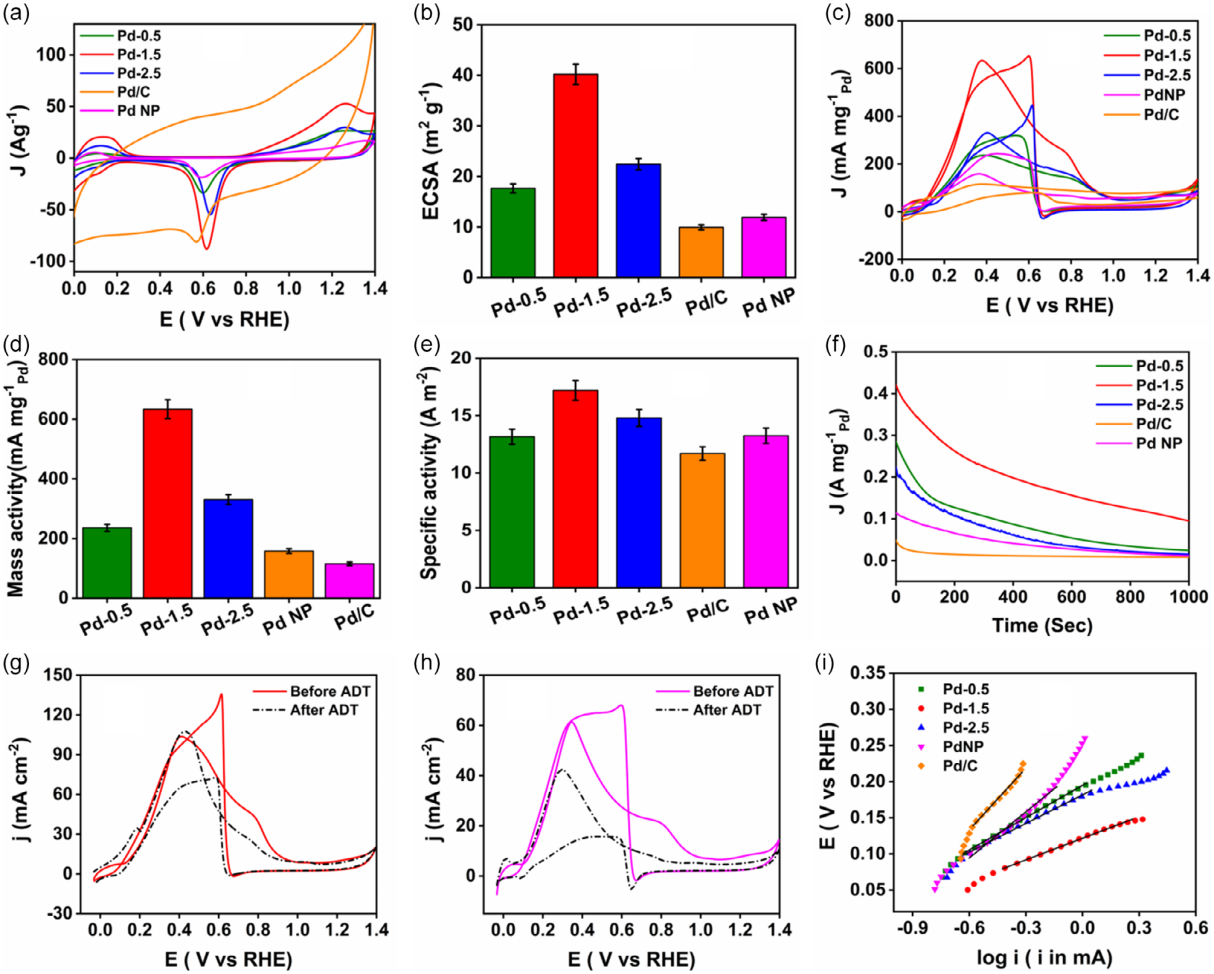
a) Plot of ECSA by PdO reduction method in N_2_ saturated 0.5 m H_2_SO_4_ at a scan rate of 50 mV s^−1^; b) bar diagram of calculated ECSA; c) mass normalized catalytic activity in 0.5 m HCOOH + 0.5 m H_2_SO_4_ at a scan rate of 50 mV s^−1^; d) bar diagram of mass activity; e) bar diagram of specific activity; f) Comparison of chronoamperometric stabilities at 0.1 V; ADT test for g) Pd‐1.5, and h) PdNP; and i) Tafel plot of different nanostructures.

The active surface area accounts for the total number of reaction active site positions per unit area of the catalyst. Determination of the electrochemically active surface area (ECSA) is necessary to assess the intrinsic activity of the Pd‐Au‐Ag nanostructures toward the FAOR, which is affected by the specific geometry and electronic structure of active sites.^[^ ^46^ ^]^ **Figure** **4**a shows the cyclic voltammetry of the nanoparticles in N_2_‐saturated 0.5 m H_2_SO_4_ at a scan rate of 50 mV s^−1^. The peaks below 0.4 V during both scans correspond to the hydrogen adsorption/desorption region. The peaks that appeared above 0.8 V in the forward scan and around 0.5–0.7 V in the reverse scan can be ascribed, respectively, to the oxidation of the Pd surface and the reduction of the thus‐formed PdO layer.^[^ ^47^ ^]^ Since carbon acts as a good capacitive material, due to high carbon content in 10 wt% Pd/C, it shows a large capacitive current in the CV as shown in Figure 4a (orange curve). As other materials do not contain carbon, they show a prominent PdO reduction peak without a large capacitive current. In the case of Pd based catalyst, the ECSA can be measured by integrating the area under the PdO reduction peak obtained by using the formula.

(5) $${\text{ECSA(m}}^{2}{\text{ g}}^{-1})=\frac{Q_{0}}{q_{0}}\;\times \frac{1}{W}$$

Where *Q*_0_ is the average charge associated with the Pd oxide reduction peak obtained from the CV curve, *q*_0_ is the charge density associated with the reduction of a monolayer of Pd oxides, which is equal to 424 μC cm^−2^, and *W* is the metal loading.^[^ ^16^ ^]^ As observed from the bar plot shown in Figure 4b, ECSA follows the trend Pd‐1.5 > Pd‐2.5 > Pd‐0.5 > PdNP > Pd/C. ECSA of Pd‐1.5 is 3.4 and 4 times higher than PdNP and Pd/C respectively. An increased electrochemically active surface area indicates the increased number of active catalytic sites present on the nanocatalyst. As the Pd is deposited on the surface of the bimetallic prism and generates bud‐like extensions of Pd‐Ag co‐depositions as shown in Figure 2, S2, and S3, Supporting Information, it produces a rough surface which is clearly visible from the TEM images (Figure 1). Surface roughness increases from Pd‐0.5–Pd‐1.5 and due to the overdeposition of Pd the roughness again decreases for Pd‐2.5 and the edges of the particle become smooth. The ECSA of Pd‐deposited particles is higher than that of PdNP due to the lattice mismatch and surface strain generation on the particle.

The electrocatalytic activity of the as‐prepared nanoparticles for the FAOR was studied by cyclic voltammetry in N_2_‐purged H_2_SO_4_ solution. Figure 4c shows the mass (Pd) normalized CVs of the trimetallic prisms, PdNP, and commercial 10 wt% Pd/C catalyst at 50 mV s^−1^ scan rate in the potential window of 0–1.4 V (vs RHE). During the positive scan, a large anodic peak is observed for all the catalysts in the potential range of 0.2–0.5 V, which is due to the formic acid oxidation via the direct pathway (dehydrogenation). A faint hump is observed around 0.80 V which corresponds to the indirect pathway (dehydration) which is negligible in our case.^[^ ^48^ ^]^ In the reverse scan of CV, the current density again increases and gives an anodic peak which is due to the further oxidation of the HCOOH molecule on the regenerated catalyst surface obtained in situ after the reduction of the so‐formed Pd‐O layer. Thus CV profile gives a clear indication that the FAO reaction proceeds via a direct pathway with negligible CO adsorption on the synthesized NPs. From the CV curve, two aspects bring interest to the Pd‐1.5 NP. First, the onset potential of the Pd‐1.5 NP is the lowest among all other synthesized NPs. Second, the current density of FA oxidation in both forward and reverse potential sweep is much higher than other catalysts including Pd/C. Since the current density and onset potential are the two most important parameters for a quantitative assessment of an electrocatalyst for its commercialization, higher current density and lower onset potential make Pd‐1.5 NP the best catalyst among the synthesized other NPs. No anodic peak is observed for the Au‐Ag bimetallic prism in the forward scan as shown in (Figure S5, Supporting Information) indicating that the Au‐Ag bimetallic prism has no electrocatalytic activity for the formic acid electrooxidation.^[^ ^41^ ^]^ The mass activity of the NPs is shown in a bar plot in the Figure 4d. As observed, the mass activity increases in the order Pd‐1.5 > Pd‐2.5 > Pd‐0.5 > PdNP > Pd/C. The mass activity of Pd‐1.5 is 4.5 times higher than PdNP and 7 times higher than Pd/C. As Pd‐1.5 has the highest ECSA and a suitable lattice strain is generated in the particle, a synergistic effect influences its activity to make it the best catalyst among all the synthesized nanoparticles. Though Pd is an effective metal for HCOOH oxidation, the calculated microstrain from the Scherrer equation for PdNP (9.3 × 10^−3^ radian) is less than that of Pd‐1.5 (14.08 × 10^−3^ radian) making PdNP a less effective catalyst in comparison with Pd‐1.5. Figure 4e reveals that the specific activity of the catalysts for FAOR at a potential of 0.5 V follows the order: Pd‐1.5 (20.7 A m^−2^) > Pd‐2.5 (17.9 A m^−2^) > Pd‐0.5 (14.1 A m^−2^) > PdNP (13.4 A m^−2^) > Pd/C (10.4 A m^−2^). The trend of specific activity follows a similar trend to that of mass activity as shown in Figure 4d. Comparing the performance with the PdNP and Pd/C the apparent improvement of the intrinsic activity was found for the Pd‐deposited trimetallic prism nanoparticles where lattice strain plays a crucial role. The formation of true and mixed alloy heterostructure of Ag‐Pd introduces tensile strain in the Pd lattice due to the mismatch of lattice constant between Ag (4.079 Å) and Pd (3.859 Å). When the tensile strain occurs, the width of the d‐band and the energy of its center will be altered, leading to a change in the electronic properties of the surface, chemisorption energy, and activation barrier of the reaction. In tensile strain, the atoms are pulled apart, and the average coordination number decreases, leading to the reduced overlap of the d orbitals keeping the same degree of d‐band filling, and consequently, the band narrows. As a result, the upshift of the d‐band center increases the density of states (DOS) at the Fermi level, which lowers the binding energy of electrons and makes the Pd surface easier to ionize. Consequently, the bond strength between Pd and intermediate species COOH_ads_ increases and the catalytic activity by promoting dehydrogenation of FA is enhanced.^[^ ^49^, ^50^, ^51^ ^]^ Meanwhile, the introduction of Ag can accelerate the oxidation of the poisonous intermediate (CO) and consequently inhibit the poisoning of the Pd active sites.^[^ ^11^ ^]^ Pd is not only deposited at the edge of the prism but also it is deposited on the surface of the Au‐Ag bimetallic prism template from where the Ag atoms are leached out. So, those surface Pd also suffer from tensile strain due to a lattice mismatch between Au and Pd and positively contribute to FAO. As the electronegativity of Au is higher than Pd, surface Pd becomes more in an oxide state and it becomes more reluctant towards surface oxide species formation. So there will be less chance of blocking the active Pd surface by the intermediate oxide species in the course of the reaction maintaining higher catalytic activity.

The stability of the catalysts was investigated by chronoamperometric studies in 0.5 m H_2_SO_4_ + 0.5 m HCOOH at 0.1 V for 1000 s. Due to the adsorption of intermediate carbonaceous species to the Pd sites during the oxidation of FA, the current density of all prepared catalysts decayed rapidly in the initial period and stabilized after a certain period. Out of all prepared trimetallic catalysts, Pd‐1.5 exhibits higher steady‐state current density as shown in Figure 4f. It is observed that the current densities of Pd‐1.5, Pd‐0.5, Pd‐2.5, PdNP, and Pd/C are 95.3, 24.4, 14.9, 11.0, and 8.2 mA mg^−1^ respectively after 1000 s. This result suggests the highest stability of the Pd‐1.5 particle which is 8.5 and 11.6 times higher than PdNP and Pd/C respectively. So, the spatial, orientational, and electronic support of the Au‐Ag prism could have a synergistic effect on improving the durability of the catalyst. Moreover, increased lattice strain offers better electrocatalytic stability towards FAOR. The accelerated durability test further confirms the remarkable stability of the Pd‐1.5 than PdNP as shown in Figure 4g,h. After 500 cycles of ADTs, there is only a little change in peak current density for Pd‐1.5 suggesting that the catalyst did not suffer an obvious poisoning phenomenon after the ADT test, and maintains a stable FAOR activity^[^ ^52^ ^]^ while in the case of PdNP, we observed a 33% decrease in peak current density.

The kinetics of the FAOR can be studied by Tafel slope investigation. The Tafel slope was determined by recording the quasi‐static measurement of linear sweep voltammetry with a low scan rate at 5 mV s^−1^ as shown in (Figure S6, Supporting Information). Tafel plots of the catalysts are shown in Figure 4i and the electrochemical control region of all Tafel plots presents a good linear relationship, which can be well fitted to the Tafel equation =a+b(logj), where *η, a, b*, and *j* are over potential, intercept, slope, and current density respectively.^[^ ^15^ ^]^ Tafel plots can be divided into two parts, (i) low and (ii) high potential regions.

(6) $$\text{HCOOH}+\text{Pd}\to \text{Pd}—\text{COOH}+H^{+}+e^{-}$$

(7) $$\text{Pd}—\text{COOH}\to \text{Pd}+{\text{CO}}_{2}+H^{+}+e^{-}$$

(8) $$\text{Pd}+H_{2}O\to \text{Pd}—\text{OH}+H^{+}+e^{-}$$

(9) $$\text{Pd}—\text{OH}+\text{Pd}—\text{COOH}\to 2\text{Pd}+{\text{CO}}_{2}+H_{2}O$$

The overall FAO reaction is explained above. In the low overpotential region the oxidation of formic acid proceeds via (6) and (7) pathways whereas in the high potential region the oxidation reaction proceeds via (8) and (9) pathways. At low potential with a low scan rate, path (6) is the rate‐determining step.^[^ ^17^ ^]^ A Tafel slope value lower than 120 mV dec^−1^ indicates the primary path where dehydrogenation and formate formation is the rate‐determining step and a value lower than 120 mV dec^−1^ proposes enhanced dehydrogenation mainly because of fewer poisoning species.^[^ ^53^, ^54^ ^]^ The obtained Tafel slopes of different catalysts are Pd‐0.5(148 mV dec^−1^), Pd‐1.5 (91 mV dec^−1^), Pd‐2.5 (130 mV dec^−1^), PdNP (212 mV dec^−1^), and Pd/C (284 mV dec^−1^). The lowest value of the Tafel slope for Pd‐1.5 indicates the faster charge transfer kinetics and highest intrinsic catalytic activity towards FAO reaction. Additionally, Pd‐1.5 exhibits a higher output current density than all other synthesized catalysts at the same potential region.

**Figure 5:**
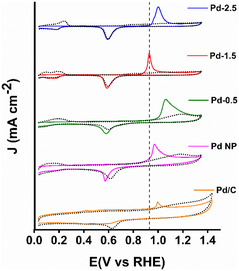
CO stripping voltammogram of different catalysts in 0.5 m H_2_SO_4_ at a scan rate of 50 mV s^−1^.

A further investigation and evaluation of the antipoisoning ability is a significant aspect of the high‐performance catalysts. To assess the poisoning effect of CO on our synthesized catalysts, we have performed the voltammetric CO stripping experiments, i.e., the oxidation of an adsorbed CO submonolayer on the catalyst surface. Notably, the voltammetric stripping of a CO monolayer is highly sensitive to surface monolayer and structure. The typical CO stripping voltammogram is shown in **Figure** **5** for different nanocatalysts. In the first cycle (solid line) the absence of the H_des_ peak in the low potential region indicates that CO was successfully adsorbed on the catalytic surface and there is a CO oxidation peak at the higher potential. In the next cycle (dotted line), the CO oxidation peak disappeared and at the same time, the recovery of the H_des_ peak indicates that the CO adsorbed on the catalyst surface is completely oxidized and eliminated.^[^ ^55^, ^56^ ^]^ According to the CO stripping voltammogram, the lower the CO oxidation onset potential, the higher the antipoisoning ability. From the zoomed image shown in (Figure S7, Supporting Information) the onset potential of different catalysts as Pd‐0.5, Pd‐1.5, Pd‐2.5, PdNP, and Pd/C are identified as 1.07, 0.85, 0.92, 0.99 and 0.97 V respectively. The result shows that Pd‐1.5 has the least onset potential which is 80 and 100 mV negatively shifted with respect to PdNP and Pd/C respectively, manifesting that Pd‐1.5 possesses a better CO_ads_ intermediate tolerance capability towards FAOR. The superiority of Pd‐1.5 compared to other synthesized monometallic, bimetallic, and trimetallic nanoparticles is clearly understandable by comparing different electrochemical parameters as shown in **Table** **3**.

**Table 3:** Experimentally obtained different electrochemical parameters for our synthesized nanoparticles.

Nanoparticle	ECSA [m^2^ g^−1^]	MA [mA mg^−1^_Pd_]	SA [A m^2^]	Tafel slope [mV dec^−1^]	CO oxidation onset potential [V]
Pd‐0.5	17.6	236	14.1	148	1.07
Pd‐1.5	40.2	634	20.7	91	0.85
Pd‐2.5	22.4	331	17.9	130	0.92
PdNP	11.9	158	13.4	212	0.99
Pd/C	9.9	115	10.4	284	0.97

## 4 Conclusion

In summary, the Pd‐lined strained Au‐Ag‐Pd trimetallic nanoparticles with different thicknesses of Pd‐deposition at the edges of the bimetallic prism particle were successfully synthesized by galvanic replacement and co‐reduction of Ag and Pd. In the synthesized unique trimetallic heterostructure, a certain percentage of Ag forms an alloy with Pd and generates lattice strain in the particle due to lattice mismatch between Ag and Pd. This study shows that the thickness of the Pd layer can be increased up to a certain nanometer after which self‐nucleation of Pd atoms starts to form individual Pd particles. Moreover, the surface of the over‐deposited particle becomes smooth which decreases the ECSA and hence catalytic activity. Though Pd is the effective metal for HCOOH reduction, the Au‐Ag prism template gives structural stability and the synergistic effect of Au and Ag enhances the catalytic activity of the trimetallic heterostructure towards FAOR by following the dehydrogenation pathway. The suitable lattice strain resulting from the mismatch between the Ag/Pd and Au/Pd leads to the change of electronic structure and the d‐band center of the Pd particle, which could adjust the adsorption strength of formic acid molecule and the intermediate species to boost the catalytic activity. Overall this study opens up the opportunity to synthesize a unique trimetallic heterostructure using different reduction potentials of the used metals to get the desired tuned properties for a particular electrocatalytic oxidation or reduction reaction with their potential application in low‐temperature fuel cells.

## Conflict of Interest

The authors declare no conflict of interest.

## Author Contributions

**Dulal Senapati**: conceptualization: (lead); data curation: (equal); formal analysis: (equal); investigation: (lead); methodology: (lead); supervision: (lead); writing—original draft: (equal); writing—review & editing: (lead). **Sourav Mondal**: conceptualization: (lead); data curation: (lead); formal analysis: (lead); investigation: (equal); methodology: (equal); writing—original draft: (equal). **Sandip Kumar De**: conceptualization: (equal); formal analysis: (supporting); methodology: (supporting). **Tanmay Ghosh**: data curation: (supporting); investigation: (supporting). **Subrata Mondal**: formal analysis: (supporting); methodology: (supporting). **Mihir Manna**: formal analysis: (supporting); validation: (supporting); writing—review & editing: (supporting).

## Supporting information

Supplementary Material

## Data Availability

The data that support the findings of this study are available from the corresponding author upon reasonable request.
