# Supplementary material for: Pd‐Lined Strained Trimetallic Au‐Ag‐Pd Nanoprism for Enhanced Electrocatalytic Activity Towards Formic Acid Oxidation
Source: Small Sci. 2025 Apr 15;5(8):2500063. doi: 10.1002/smsc.202500063 (PMC12362725; doi:10.1002/smsc.202500063)
Supplement: Supplementary file 1 — Supplementary Material [file SMSC-5-2500063-s001.pdf]

Supporting Information

Pd-lined Strained Trimetallic Au-Ag-Pd Nanoprism for Enhanced Electrocatalytic Activity towards Formic Acid Oxidation

Sourav Mondal[1], Sandip Kumar De[2], Tanmay Ghosh[3], Subrata Mondal[4], Mihir Manna[1], Dulal Senapati[1,*]

[1]Chemical Sciences Division, Homi Bhabha National Institute, Saha Institute of Nuclear Physics, A CI of Homi Bhabha National Institute, 1/AF Bidhannagar, Kolkata 700064, India.

[2]Department of Chemistry, SRICT-ISR, UPL University of Sustainable Technology, Gujarat 393135, India.

[3]Institute of Materials Research and Engineering (IMRE), Agency for Science, Technology and Research (A*STAR), 2 Fusionopolis Way, Innovis # 08-03, Singapore 138634, Republic of Singapore.

[4]Department of Chemistry, Dinhata College, Dinhata, Cooch Behar 736135, India

*dulal.senapati@saha.ac.in

Table of Contents

**Section Headings**	**Page Numbers**
Optical and electronic characterization of nanoparticle	S2
Time-dependent Pd-Ag co-deposition on the edge of the bimetallic prism	S2
Co-deposition of Pd and Ag on the edges of the bimetallic prism at room temperature (27^0C)	S3
High-resolution XPS for C1s, Ag 3d, and Au 4f of different nanoparticles	S3-S4
Catalytic activity of Au-Ag bimetallic prism for FAOR	S4

Linear sweep Voltammetry of different catalysts	S4
CO stripping voltammetry	S5

1. Optical and electronic characterization of nanoparticle

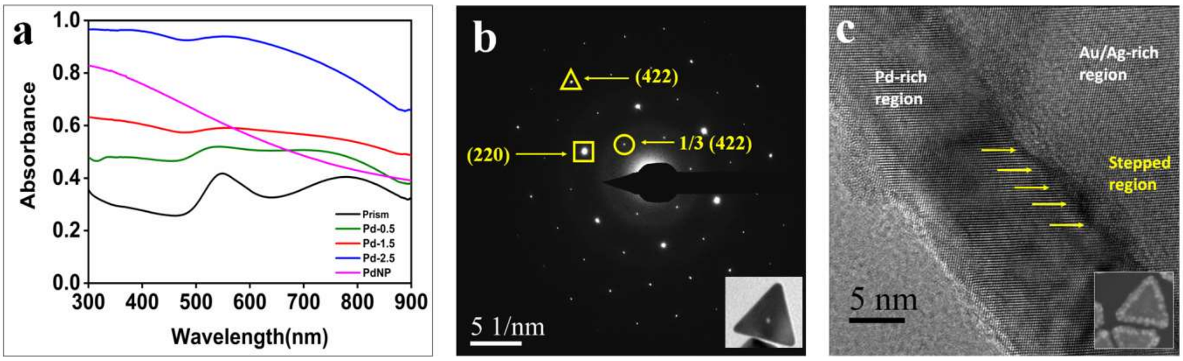

Figure S1: (a) UV-Vis absorption spectra of the bimetallic prism, Pd-0.5, Pd-1.5, Pd-2.5, and monometallic PdNP, (b) SAED pattern of bimetallic Au-Ag nanoprism (inset: TEM of a single bimetallic Au-Ag nanoprism), (c) HRTEM of the stepped regions at the interface between Pd-layer and Au-Ag basal plane for Pd-0.5 (inset: SEM of a single trimetallic nanoprism).

2. Time-dependent Pd-Ag co-deposition on the edge of the bimetallic prism

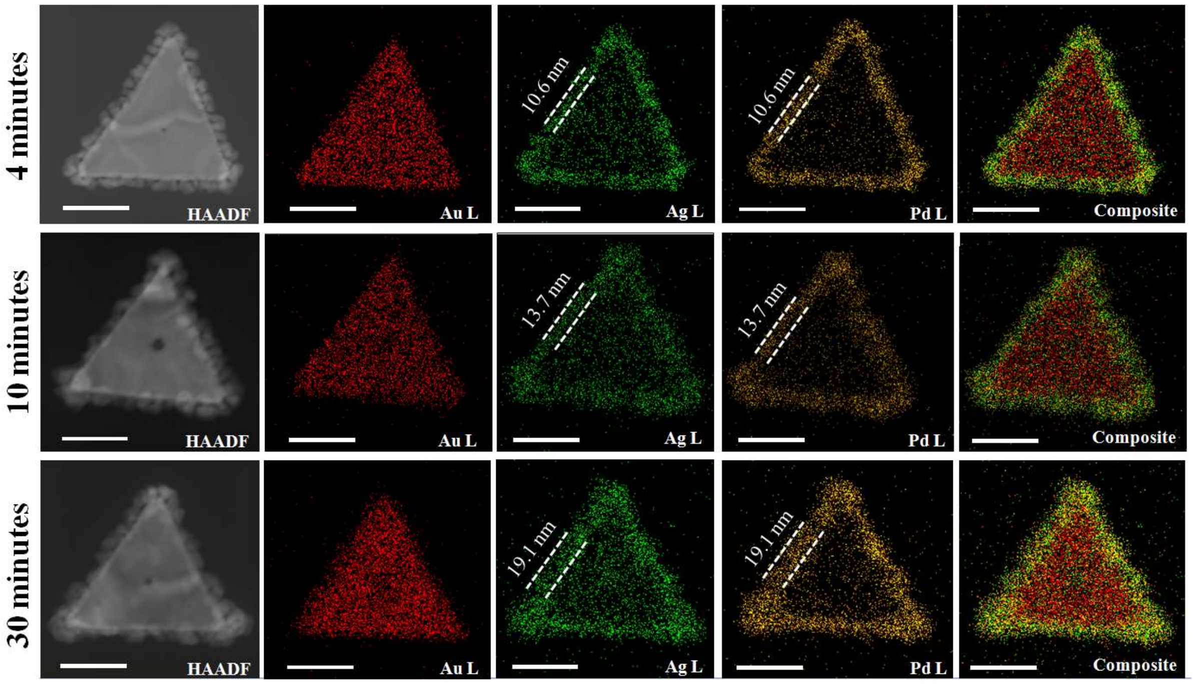

Figure S2: HAADF and EDX elemental mapping of Pd-1.5 nanoparticle after 4, 10, and 30 min of the addition of Pd in the 3rd step of the synthesis. The dotted lines show the thickness of the Pd-Ag deposited layer on the Au-Ag bimetallic prism.

3. Co-deposition of Pd and Ag on the edges of the bimetallic prism at room temperature (27^0C)

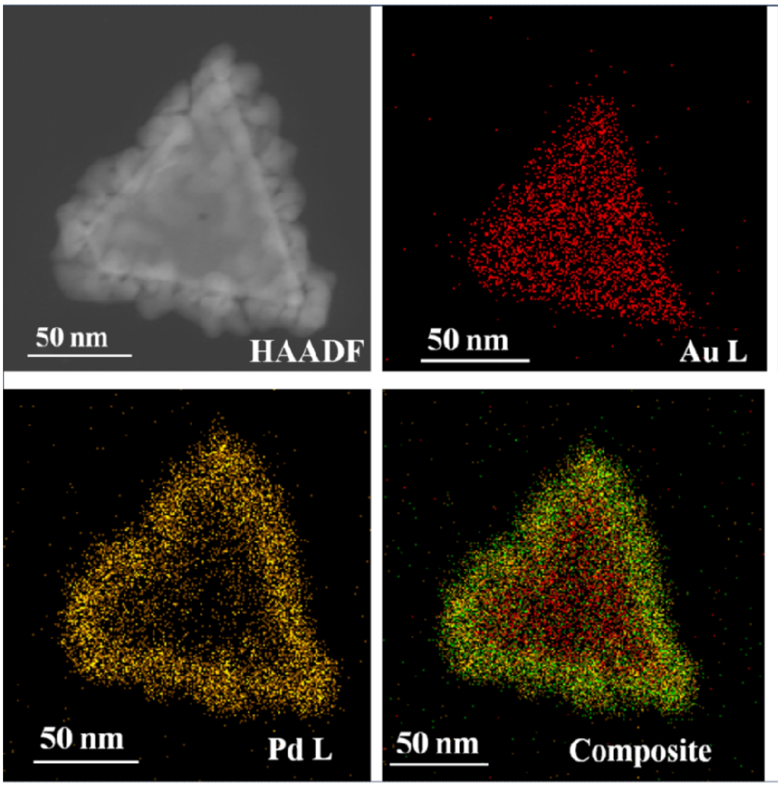

Element	Atomic percentage
AuL	2.8
AgL	8.3
PdL	88.9

Figure S3: HAADF, EDX elemental mapping, and a list of atomic percentages of different elements present in Pd-1.5, synthesized at Room Temperature.

4. High-resolution XPS for C1s, Ag 3d, and Au 4f of different nanoparticles

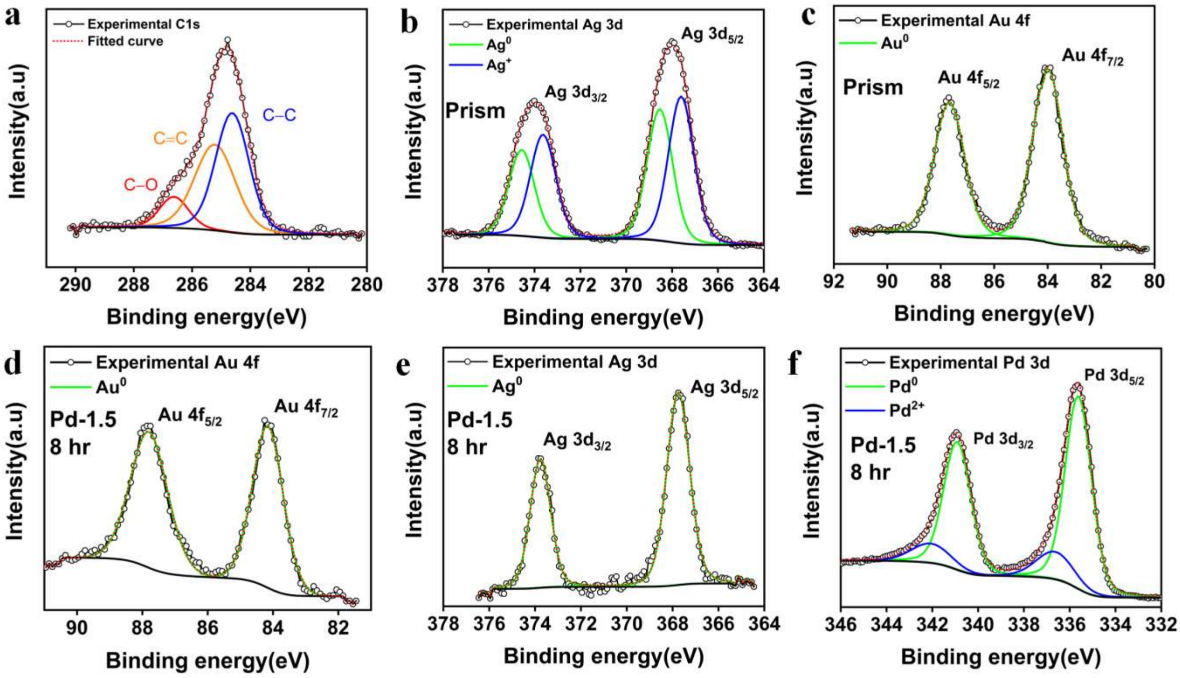

Figure S4. XPS spectra of (a) C1s in Pd-1.5 nanoparticle; (b) Ag 3d and (c) Au 4f in bimetallic prism; (d) Au 4f, (e) Ag 3d, and (f) Pd 3d in Pd-1.5 particle after 8h of chronoamperometric reaction.

5. Catalytic activity of Au-Ag bimetallic prism for FAOR

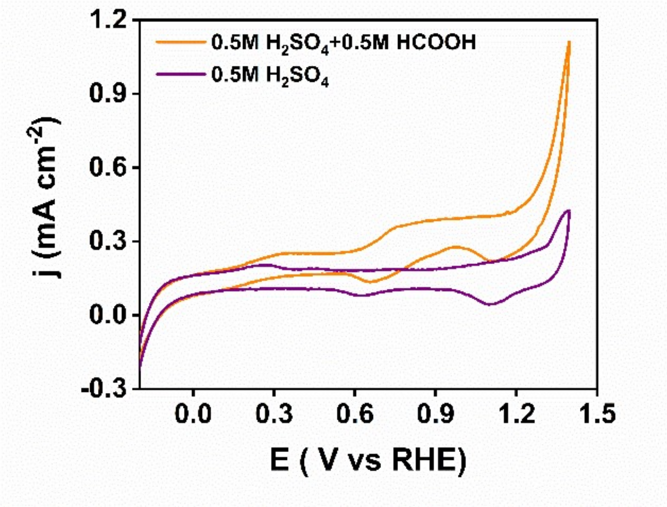

Figure S5. Cyclic voltammetry of bimetallic prism in 0.5 M H_2SO_4 with and without HCOOH at a scan rate of 50 mV s^{-1}.

6. Linear sweep Voltammetry of different catalysts

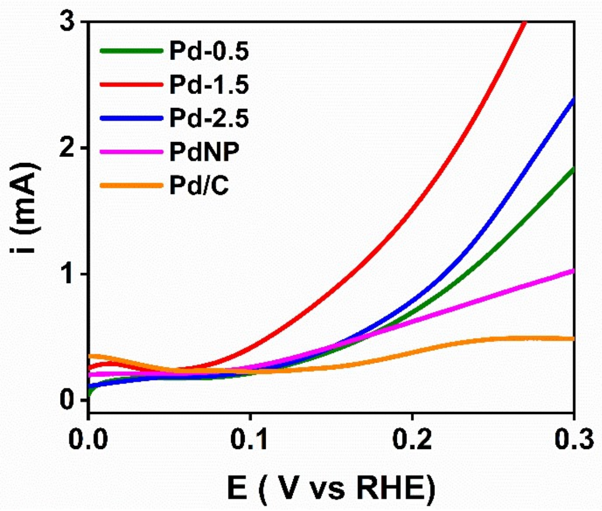

Figure S6. Enlarged image of the LSV curves in 0.5 M H_2SO_4 + 0.5 M HCOOH at a scan rate of 5 mV s^{-1}.

7. CO stripping voltammetry

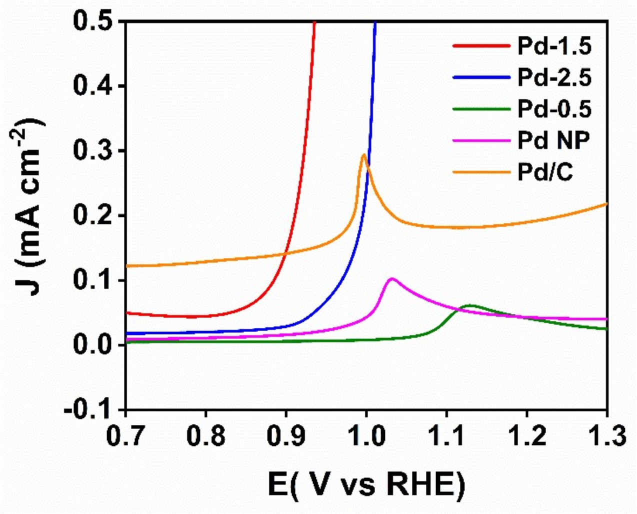

Figure S7. Zoomed image of CO stripping cyclic voltammetry in 0.5 M H_2SO_4 at a scan rate of 50 mV s^{-1}.
